# Exercise interventions for depressive, manic, and anxiety symptoms in bipolar disorder: a systematic review and meta-analysis

**DOI:** 10.3389/fpsyt.2025.1648008

**Published:** 2025-09-22

**Authors:** Xinmo Li, Feike Liu, Feng Ding, Xiaochen Ma, Yongguo Zhu

**Affiliations:** ^1^ Capital University of Physical Education and Sports, Beijing, China; ^2^ Shanghai University of Sport, Shanghai, China

**Keywords:** bipolar disorder, exercise intervention, depression, mania, anxiety, meta-analysis

## Abstract

**Background:**

To evaluate the efficacy of exercise interventions on depressive, manic, and anxiety symptoms in patients with bipolar disorder (BD) through a systematic review and meta-analysis, and to explore the impact of different exercise parameters (session duration, frequency, etc.) on these symptoms.

**Methods:**

We comprehensively searched Embase, Web of Science, PubMed, The Cochrane Library, Wan fang, Weipu Database(VIP), and China National Knowledge Infrastructure (CNKI) databases for randomized controlled trials (RCTs) published until May 2025. Included studies met the following criteria (1): participants aged ≥18 years with BD diagnosed according to Diagnostic and Statistical Manual of Mental Disorders, Fifth Edition (DSM-5) or International Classification of Diseases, 11th Revision (ICD-11) criteria (2); exercise as the primary intervention (3); control groups receiving standard rehabilitation treatment. Study quality was assessed using the Physiotherapy Evidence Database (PEDro) scale. Meta-analyses were performed using Stata 18.0, and evidence quality was evaluated with Grading of Recommendations Assessment, Development and Evaluation (GRADE) methodology.

**Results:**

A total of 7 RCTs n=576 were included. Meta-analysis demonstrated that exercise significantly improved depressive symptoms (SMD =-0.63, 95%CI: -1.11 to -0.14, P=0.01) and anxiety symptoms (SMD =-0.70, 95%CI: -1.26 to -0.15, P<0.05) in BD patients, but showed no significant effect on manic symptoms (SMD =-0.23, 95%CI: -0.67 to 0.21, P>0.05). Subgroup analyses revealed that exercise protocols featuring session duration ≤1 hour/session (SMD= -0.86, P=0.02), frequency >5 sessions/week (SMD = -0.76, P<0.01), and intervention period ≤12 weeks (SMD = -0.79, P=0.02) produced more pronounced improvements in depressive symptoms. The GRADE approach rated the quality of evidence as low for all outcomes (depression, anxiety, mania), with downgrading factors including substantial heterogeneity, imprecision, and risk of bias.

**Conclusion:**

Current low-quality evidence suggests that exercise may alleviate depressive and anxiety symptoms in BD patients (particularly with high-frequency, prolonged-duration, short-term protocols), while its effect on manic symptoms remains inconclusive. These findings should be interpreted cautiously due to methodological limitations of included studies.

**Systematic review registration:**

https://www.crd.york.ac.uk/prospero/, identifier CRD420251041926.

## 1 Introduction

Bipolar Disorder (BD) is a severe psychiatric condition characterized by recurrent episodes of mania and depression, with a global lifetime prevalence of approximately 1–2% (1). The World Health Organization identifies BD as a leading cause of disability among psychiatric disorders, ranking among the top six in disability-adjusted life years (DALYs) in the 2019 Global Burden of Disease study, with a disability weight of 0.576, surpassing most chronic physical conditions (2, 3). A large-scale survey in China revealed that the disease burden of BD is substantially underestimated, driven by low diagnosis rates (~40% of cases undiagnosed) and significant treatment gaps, particularly in low-income populations (4). Moreover, BD patients exhibit pronounced impairments in social functioning, with ~60% demonstrating reduced occupational capacity (5). BD impairs emotional regulation and is frequently associated with anxiety and social dysfunction, severely compromising quality of life and imposing a significant socioeconomic burden, with life expectancy reduced by 10 – 20 years relative to the general population (1, 6). Consequently, addressing the manic and depressive symptoms of BD is a pressing priority requiring urgent attention.

Prior studies demonstrate that pharmacological interventions (e.g., mood stabilizers, antipsychotics) and psychological therapies (e.g., cognitive behavioral therapy) are primary treatments, yet many patients experience residual symptoms, adverse reactions, or treatment resistance (7). Despite advances in pharmacological treatments for bipolar disorder (BD), approximately 70% of patients fail to achieve sustained remission, with a substantial proportion of their illness course dominated by persistent subsyndromal symptoms (8). These residual symptoms not only impair psychosocial functioning but also increase relapse risk (9). Emerging evidence suggests that exercise may help address this therapeutic gap. Exercise, a structured and repetitive subset of physical activity, is designed to enhance or maintain physical fitness (10). As a safe, cost-effective intervention with minimal adverse effects, it holds significant promise for promoting mental well-being (11–14). Exercise exerts multifaceted regulatory effects, directly mitigating mood disorders. Its mechanisms primarily involve enhancing neuroplasticity (e.g., increasing brain-derived neurotrophic factor (BDNF) release and synaptic function (15)), stabilizing hypothalamic-pituitary-adrenal (HPA) axis activity (e.g., reducing excessive cortisol secretion and stress-induced damage (2)), and modulating systemic inflammation (e.g., suppressing pro-inflammatory cytokine IL-6 while elevating anti-inflammatory IL-10 (16)). Furthermore, exercise improves physical health, and its low risk, high tolerability, and adaptable intensity render it suitable for long-term use, particularly as an adjunctive therapy for treatment-resistant BD (17). Additionally, exercise promotes patient autonomy and social engagement, shifting from a passive treatment paradigm, while remaining cost-effective and accessible (18). Compared with the adverse effects of pharmacotherapy, resource constraints of psychotherapy, and high costs of physical therapy, exercise provides preventive, therapeutic, and rehabilitative benefits, serving as a safe, accessible, and versatile intervention for mental health management (19).

Prior meta-analyses (11, 20) confirm that exercise significantly reduces core symptoms in patients with depression and anxiety disorders. However, the efficacy of exercise in ameliorating depressive, manic, and anxiety symptoms in BD remains uncertain, with conflicting findings (21). A pilot study by Legrand and Heuze reported that a high-frequency exercise group (3–5 sessions per week) exhibited significantly greater reductions in depressive symptoms at 4 and 8 weeks compared with a low-frequency control group (1 session per week), though improvements in the high-frequency group decelerated between weeks 4 and 8 (22). A meta-analyse by Schuch et al. (2016) demonstrated that aerobic exercise sessions exceeding 60 minutes significantly reduced depressive symptoms, whereas sessions of ≤30 minutes had no notable effect (11). Regular yoga practice may lower the risk of manic episodes in BD patients (23), whereas Wright et al. reported that marathon training or high-intensity interval training (HIIT) triggered manic episodes in some BD patients (24). Notably, high-quality studies on exercise interventions for BD patients during manic episodes remain scarce (25). Exercise significantly ameliorates anxiety symptoms in patients with anxiety disorders (26–28), yet its efficacy for anxiety symptoms in BD patients remains inconclusive.

A review of prior literature suggests that the efficacy of exercise interventions for BD remains uncertain, with conflicting results. Given evidence suggesting that exercise interventions may be less effective for depressive symptoms in BD compared with unipolar depression, with inconsistent dose-effect relationships for manic symptoms and limited evidence for anxiety symptom efficacy, this study evaluates the effectiveness of exercise interventions in ameliorating depressive, manic, and anxiety symptoms in BD patients and examines the dose-response relationships of exercise parameters (session duration, frequency and intervention period) to provide evidence-based guidance for personalized adjunctive BD treatment.

## 2 Materials and methods

### 2.1 Study framework

This study complies with the Preferred Reporting Items for Systematic Reviews and Meta-Analyses (PRISMA) guidelines and Cochrane Handbook standards (29, 30). This study is registered with the International Prospective Register of Systematic Reviews (PROSPERO; CRD420251041926).

### 2.2 Search Strategy

Two researchers independently searched seven databases (Embase, Web of Science, PubMed, Cochrane Library, Wanfang, Weipu Database (VIP) and China National Knowledge Infrastructure (CNKI) databases for randomized controlled trials (RCTs) evaluating exercise interventions in bipolar disorder, from database inception to May 2025, supplemented by manual reference list searches. The search strategy combined terms for “bipolar disorder,” “exercise intervention,” and “randomized controlled trial” using “OR” for synonyms and “AND” for core element intersection, ensuring comprehensive yet precise retrieval. The search string was: (bipolar disorder OR manic-depressive illness OR bipolar affective disorder OR bipolar depression OR manic episode OR manic depression) AND (exercise therapy OR exercise intervention OR aerobic exercise OR resistance training OR mind-body therapy OR physical activity) AND (randomized controlled trial OR RCT). The search strategy is detailed in **Supplementary Table 1**.

### 2.3 Inclusion and exclusion criteria

#### 2.3.1 Inclusion criteria

##### 2.3.1.1 Study design

RCTs, blinded or unblinded.

##### 2.3.1.2 Population

Adults (≥18 years) diagnosed with bipolar disorder (Type I, Type II, or unspecified) per the Diagnostic and Statistical Manual of Mental Disorders, Fifth Edition (DSM-5) or International Classification of Diseases, 11th Revision (ICD-11) criteria, in depressive, manic/hypomanic, or remission phases; studies involving patients with comorbid serious physical (e.g., cardiovascular disease, diabetes) or mental disorders (e.g., schizophrenia) required independent BD subgroup data.

##### 2.3.1.3 Intervention

Aerobic exercise (e.g., walking, running, cycling), resistance training, mind-body exercise (e.g., yoga, tai chi), or combined interventions (e.g., exercise with mindfulness or psychotherapy). Studies were included if exercise remained the dominant component (For exercise interventions in mental health research, the exercise component should constitute ≥ 60-70% of the total intervention to ensure its dominant therapeutic role (31).

##### 2.3.1.4 Comparison

Standard treatment groups receiving pharmacotherapy (e.g., mood stabilizers), psychological interventions (e.g., cognitive behavioral therapy), or both; non-exercise control groups receiving standard care, health education, or placebo interventions.

##### 2.3.1.5 Outcomes

Hamilton Depression Rating Scale (HAM-D), Montgomery-Åsberg Depression Rating Scale (MADRS), Quick Inventory of Depressive Symptomatology (QIDS), Beck Depression Inventory (BDI), Clinical Global Impressions Scale for Bipolar Disorder (CGI-BP), Depression Anxiety Stress Scales - Depression Subscale (DASS-D), Internal State Scale (ISS), Young Mania Rating Scale (YMRS), Altman Self-Rating Mania Scale (ASRM), Bech-Rafaelsen Mania Rating Scale (MAS or BRMRS) Hamilton Anxiety Rating Scale (HARS), Generalized Anxiety Disorder 7-item Scale (GAD-7).

#### 2.3.2 Exclusion criteria

Studies were excluded if they lacked sufficient detail to confirm exercise as the primary intervention component (≥60–70% of total intervention time), did not report at least two essential exercise parameters (32)(e.g., type and intensity), used active control groups involving exercise, lacked standardized outcome measures for depression, mania, or anxiety, or presented unextractable data (e.g., only in unlabeled figures or incomplete reporting).

### 2.4 Literature screening, data extraction, and quality assessment

#### 2.4.1 Study selection and data extraction

Studies were imported into EndNote 21.Automated deduplication was performed using EndNote 21, with manual verification to ensure removal of duplicates. Two researchers independently screened titles and abstracts to exclude irrelevant studies. Full texts of remaining studies were then reviewed against inclusion and exclusion criteria. Discrepancies were resolved through discussion with a third researcher to achieve consensus. Inter-rater reliability was maintained (Kappa coefficient ≥0.8).

Data were extracted using a standardized form, capturing study characteristics (author, publication year, country, sample size, design), patient details (age, sex, BD subtype [Type I/II], disease duration), intervention parameters (exercise type, intensity, frequency, session duration, total duration, implementation method), and outcome data (baseline and endpoint means ± standard deviations for depression, mania, and anxiety scores), ensuring accuracy. Data extraction was conducted independently by two researchers, with discrepancies resolved through discussion with a third researcher to achieve consensus.

#### 2.4.2 Quality assessment

Study quality was evaluated using the Physiotherapy Evidence Database (PEDro) scale (33), assessing methodological rigor across 11 criteria: eligibility criteria, random allocation, allocation concealment, baseline comparability, participant blinding, therapist blinding, outcome assessor blinding, >85% follow-up rate, intention-to-treat analysis, between-group statistical comparisons, and point estimates with variability measures. Each criterion met scored 1 point (otherwise 0), with total scores (out of 10) classified as low (<4), moderate (4–5), good (6–8), or high quality (9–10).Only studies of moderate or higher quality were included (34).

Grading of Recommendations Assessment, Development and Evaluation (GRADE) (35) methodology was also used to evaluate evidence quality for each outcome, classified as high, moderate, low, or very low (35, 36). Downgrading factors included risk of bias (e.g., inadequate allocation concealment), inconsistency (I² >50%), indirectness (e.g., population mismatch), imprecision (wide confidence intervals), and publication bias, each potentially reducing evidence quality by 1–2 levels. Upgrading factors included large effect sizes (RR >2 or<0.5), dose-response relationships, and residual confounding, each potentially increasing evidence quality by 1 level. Evidence quality was classified as high (reliable), moderate (potentially changeable), low (likely changeable), or very low (highly uncertain).This evaluation provides robust evidence for clinical decision-making. Quality assessments were performed independently by two researchers, with discrepancies resolved through discussion with a third researcher to achieve consensus (37).

### 2.5 Data analysis

Meta-analyses were performed using Stata 18.0. When outcome measure directions varied across studies, means were multiplied by -1 to standardize effect size calculations. Effect sizes were computed using Hedges’ g with 95% confidence intervals (CI).Heterogeneity was evaluated using Cochran’s Q test and I² statistic; P< 0.1 and I² > 50% indicated heterogeneity, necessitating a random-effects model, whereas homogeneity prompted a fixed-effects model. When heterogeneity was detected (P< 0.1, I² > 50%), sensitivity and subgroup analyses were performed to investigate sources (e.g., exercise parameters, patient characteristics). Results reported effect sizes with 95% CI, with P< 0.05 deemed statistically significant. For studies using multiple scales for the same symptom, subgroup data were pooled to compute a standard deviation (SD) using the formula:


$$SD=\sqrt{\frac{\left(N_{1}-1\right)SD_{1}^{2}+\left(N_{2}-1\right)SD_{2}^{2}}{N_{1}+N_{2}-2}+\frac{N_{1}N_{2}}{N_{1}+N_{2}}\left(M_{1}^{2}+M_{2}^{2}-2M_{1}M_{2}\right)}$$

Where N_1_ , N_2_ are subgroup sample sizes, SD_1_ , SD_2_ are subgroup standard deviations, and M_1_ , M_2_ are subgroup means (30).

## 3 Results

### 3.1 Literature search results

**Figure 1:**
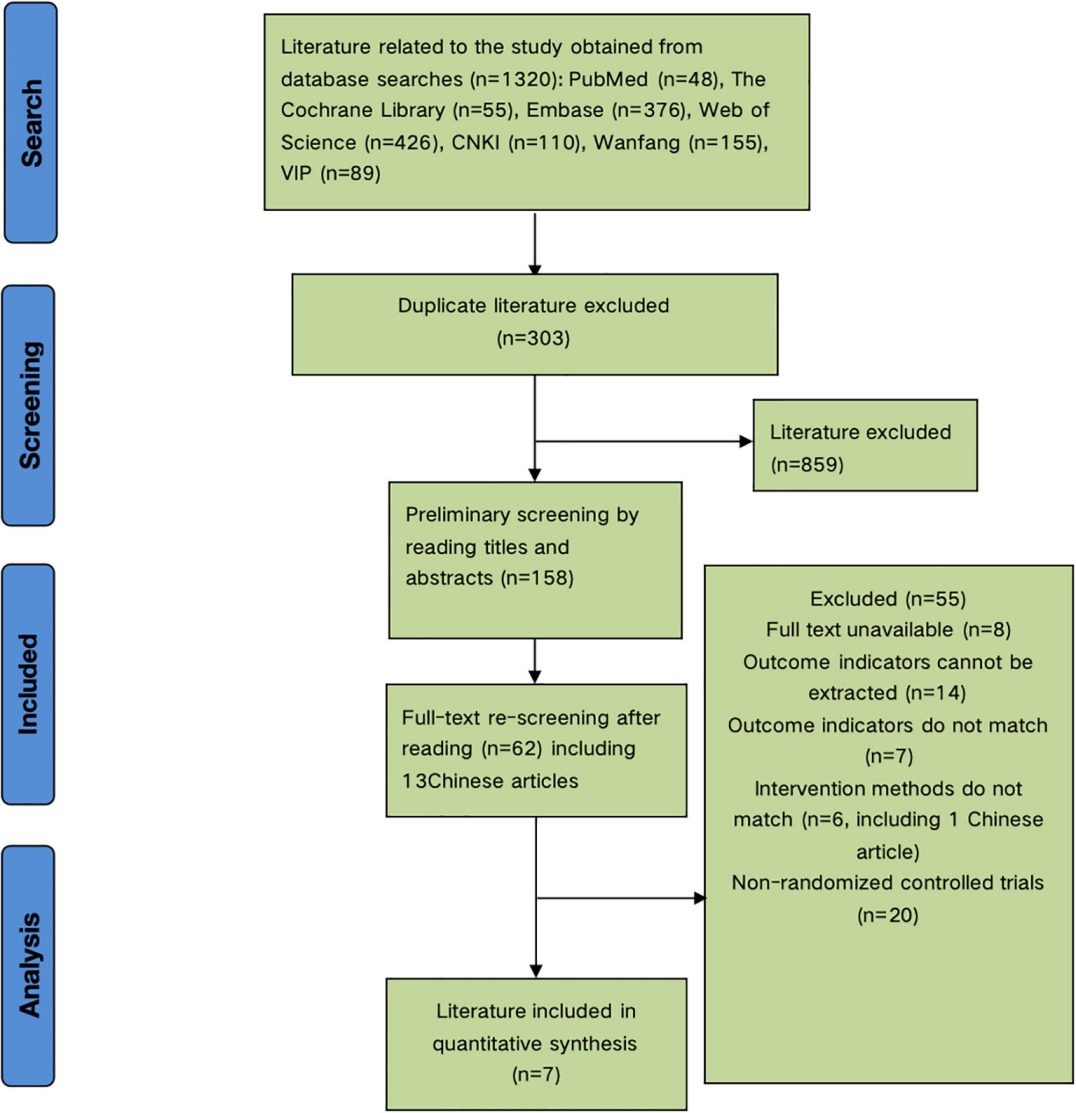
PRISMA flow diagram of study selection process.

The initial search yielded 1,320 articles. After removing 303 duplicates, 859 articles were excluded during title and abstract screening, leaving 158 articles. Full-text review excluded 8 irretrievable articles, 14 lacking relevant outcome data, 7 with non-compliant outcomes, 6 with ineligible interventions, and 20 non-randomized controlled trials. Seven articles were included in the meta-analysis (**Figure 1**).

### 3.2 Characteristics of included studies

**Table 1** summarizes the characteristics of the 7 included studies (21, 38–43), involving 576 participants (288 experimental, 288 control).Studies were published from 2012 to 2024. All studies used exercise as the experimental intervention and standard care as the control intervention. Exercise types included jogging, walking, and mixed modalities, with durations of 3–24 weeks, frequencies of 2–12 sessions per week, and session lengths of 30–120 minutes.

**Table 1:** Characteristics of included studies.

Study	Sample Size (T/C)	Gender (M/F) (T/C)	Age (years) (T/C)	Disease Duration (years) (T/C)	Intervention (T/C)	Intervention Parameters	Assessment Tools*	Outcomes
Kilbourne 2012 (38)	32/33	14/18 vs 12/21	47.3 ± 11.8 vs 43.5 ± 13.6	NR	Low-intensity exercise vs usual care	120min/session,4 sessions/week, 24 weeks	⑦	Depression: NS between-group; within-group improvement without statistical significance
Zhao 2023 (39)	50/50	32/18 vs 30/20	40.3 ± 4.2 vs 40.1 ± 4.4	3.2 ± 0.5 vs 3.1 ± 0.7	Mindfulness + moderate exercise vs usual care	30 min/session, 14 sessions/week, 4 weeks	①⑬	Depression: Significant within- and between-group improvement Anxiety: Significant within- and between-group improvement
Park 2021 (40)	11/12	2/9 vs 6/6	55.0 ± 11.3 vs 61.0 ± 6.5	NR	Low-intensity qigong vs usual care	60 min/session, 12 sessions/week, 12 weeks	②③⑧⑨	Depression: NS between-group; significant within-group improvement Anxiety: NS between-group Mania: NS between-group
Sylvia 2019 (21)	19/19	6/13 vs 7/12	39.7 ± 12.5 vs 44.3 ± 11.9	42.0 ± 12.3 vs NR	Moderate stair climbing vs usual care	30 min/session, 5sessions/week, 20 weeks	②⑤⑧⑪	Depression: Significant within-group, NS between-group Mania: Within-group improvement without statistical significance, NS between-group
Weinstock 2016 (41)	10/8	5/5 vs 3/5	41.3 ± 14.3 vs 33.6 ± 13.3	NR	Low-intensity yoga vs usual care	80 min/session, 2sessions/week, 10 weeks	③⑨⑫	Depression: Significant within-group, NS between-group Mania: Significant between-group improvement
Wang 2017 (42)	121/121	60/61 vs 63/58	42.6 ± 12.4 vs 45.1 ± 13.2	6.2 ± 3.1 vs 5.2 ± 2.2	Low-intensity calisthenics/jogging vs usual care	50 min/session, 2-3sessions/week, 12 weeks	①	Depression: Significant within- and between-group improvement
Zhang 2024 (43)	45/45	25/20 vs 23/22	45.3 ± 5.3 vs 43.5 ± 5.3	NR	Multimodal intervention vs usual care	3-week program	①②⑩⑬⑭	Depression: Significant within- and between-group improvement Anxiety: Significant within- and between-group improvement Mania: Significant within- and between-group improvement

① Hamilton Depression Rating Scale (HAMD/HDRS). ② Montgomery-Åsberg Depression Rating Scale (MADRS). ③ Quick Inventory of Depressive Symptomatology (QIDS). ④ Beck Depression Inventory (BDI). ⑤ Clinical Global Impression-Bipolar Version, Depression Subscale (CGI-BP-D). ⑥ Depression Anxiety Stress Scales, Depression Subscale (DASS-D). ⑦ Internal State Scale, Depression Subscale (ISS-D) Manic Symptoms. ⑧ Young Mania Rating Scale (YMRS). ⑨ Altman Self-Rating Mania Scale (ASRM). ⑩ Bech-Rafaelsen Mania Rating Scale (BRMRS). ⑪ Clinical Global Impression-Bipolar Version, Mania Subscale (CGI-BP-M). ⑫ Internal State Scale, Activation Subscale (ISS-A) Anxiety Symptoms. ⑬ Hamilton Anxiety Rating Scale (HAMA/HARS). ⑭ Generalized Anxiety Disorder 7-item Scale (GAD-7). NR, Not Reported; NS, Not Statistically Significant; T/C, Treatment/Control Group. Statistical Notation, Data presented as Mean ± SD unless otherwise specified. Between-group differences tested at P< 0.05 significance level.

### 3.3 Quality assessment of included studies

All 7 included studies met criteria for random allocation, baseline comparability, between-group statistical comparisons, and point estimates with variability measures.

Four studies met criteria for outcome assessor blinding and >85% follow-up rate, two for allocation concealment, and one for participant blinding. PEDro scores ranged from 6 to 8 (mean: 6.29). No low-quality studies were included, indicating good methodological quality (**Table 2**).

**Table 2:** PEDro quality assessment of included studies.

Study	Eligibility Criteria	Random Allocation	Allocation Concealment	Baseline Similarity	Subject Blinding	Therapist Blinding	Assessor Blinding	≥85% Follow-up	Intention-to-Treat	Between-group Comparison	Point Estimates & Variability	Total Score
Kilbourne 2012 (38)	1	1	1	1	0	0	1	1	0	1	1	7
Zhao 2023 (39)	1	1	0	1	0	0	0	1	1	1	1	6
Park 2022 (40)	1	1	1	1	1	0	1	0	1	1	1	8
Sylvia 2020 (21)	1	1	0	1	0	0	1	0	1	1	1	6
Weinstock 2016 (41)	1	1	0	1	0	0	1	1	0	1	1	6
Wang 2017 (42)	1	1	0	1	0	0	0	1	1	1	1	6
Zhang 2024 (43)	1	1	0	1	0	0	0	1	1	1	1	6
Total	7	7	2	7	1	0	4	4	5	7	7	44

### 3.4 Meta-analysis results

#### 3.4.1 Effects of exercise interventions on depressive, manic, and anxiety symptoms in bipolar disorder

**Figure 2:**
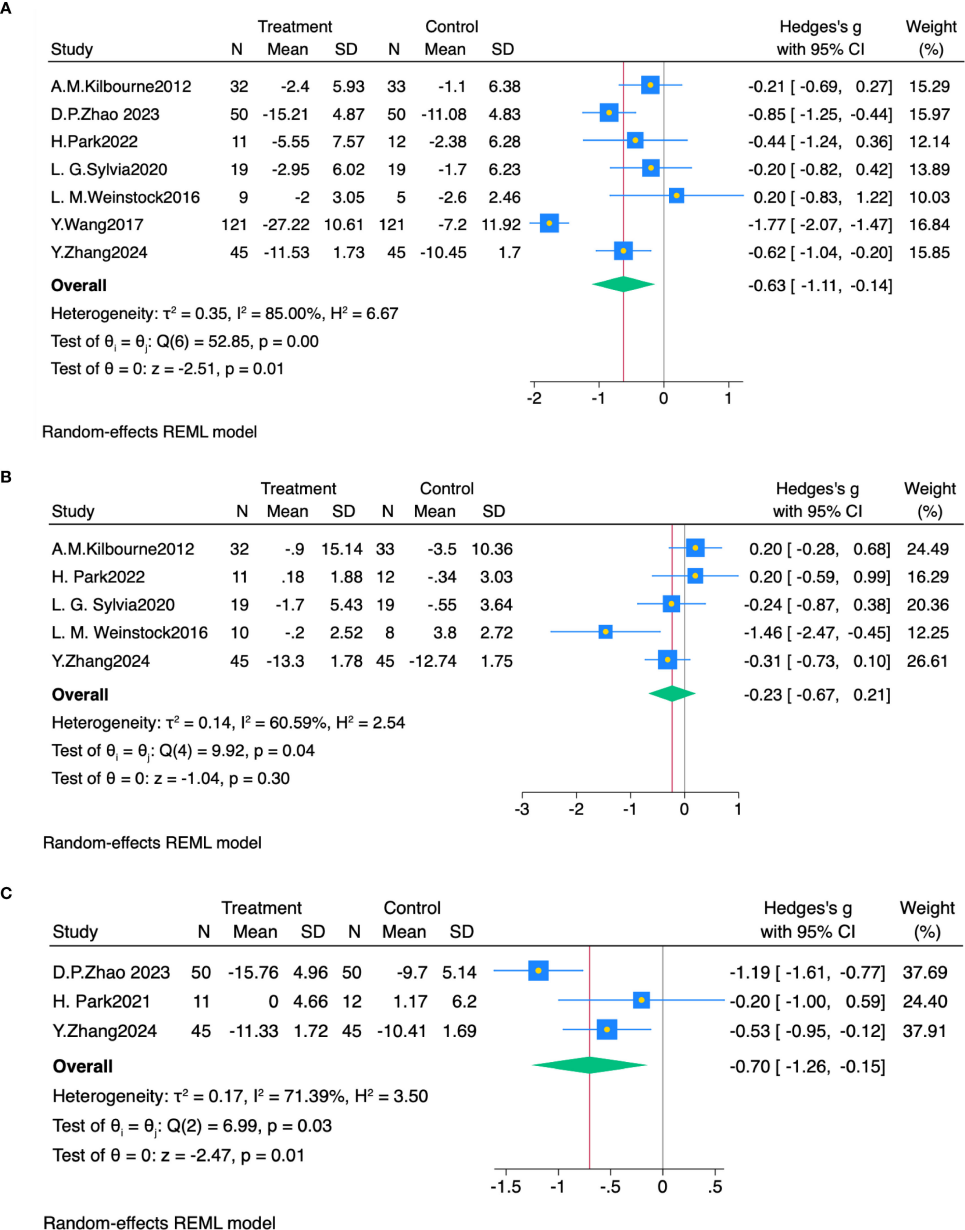
**(A)** Forest plot of exercise intervention effects on depression. **(B)** Forest plot of exercise intervention effects on mania symptoms. **(C)** Forest plot of exercise intervention effects on anxiety symptoms.

Seven studies (21, 38–43) reported depression outcomes. Random-effects meta-analysis revealed that exercise significantly ameliorated depressive symptoms in bipolar disorder (SMD = -0.63, 95% CI: -1.11 to -0.14, P< 0.05, I² = 85%), compared with controls (**Figure 2A**).

Five studies (21, 38, 40, 41, 43) reported manic symptom outcomes. Random-effects meta-analysis showed no significant effect of aerobic exercise on manic symptoms in bipolar disorder (SMD = -0.23, 95% CI: -0.67 to 0.21, P > 0.05, I² = 60.6%), compared with controls (**Figure 2B**).

Three studies (39, 40, 43) reported anxiety symptom outcomes. Random-effects meta-analysis revealed that exercise significantly ameliorated anxiety symptoms in bipolar disorder (SMD = -0.70, 95% CI: -1.26 to -0.15, P< 0.05, I² = 71.4%), compared with controls (**Figure 2C**).

#### 3.4.2 Subgroup analysis of moderating effects

Subgroup analyses explored heterogeneity sources for depressive symptoms, examining patient age, exercise duration, frequency, and intervention period (**Table 3**). Other variables, such as exercise intensity, were consistent across studies, precluding subgroup analysis. Age was categorized as 33–40 and 41–64 years; exercise duration as ≤1 h and >1 h per session; frequency as ≤5 and >5 sessions per week; and intervention period as ≤12 and >12 weeks.

**Table 3:** Results of subgroup analyses for depression symptoms.

Subgroup	Number of Studies (Participants)	I² (%)	SMD (95% CI)	P-value
Overall Effect	7 (576) (21, 38–43)	85.0	SMD = -0.63 (-1.11, -0.14)	0.01
Age Group				
- 33–40 years	2 (138) (21, 39)	65.3	SMD = -0.57 (-1.19, 0.06)	0.08
- 41–64 years	5 (438) (38, 40–43)	88.4	SMD = -0.64 (-1.31, 0.04)	0.06
Session Duration				
- >1 hour/session	2 (79) (38, 41)	0.0	SMD = -0.14 (-0.57, 0.30)	0.54
- ≤1 hour/session	4 (407) (21, 39, 40, 42)	88.4	SMD = -0.86 (-1.57, -0.16)	0.02
Frequency				
- >5 sessions/week	2 (123) (39, 40)	0.0	SMD = -0.76 (-1.12, -0.40)	<0.01
- ≤5 sessions/week	4 (363) (21, 41, 42)	91.1	SMD = -0.56 (-1.45, 0.33)	0.22
Intervention period				
- >12 weeks	2 (103) (21, 38)	0.0	SMD = -0.21 (-0.59, 0.18)	0.29
- ≤12 weeks	5 (383) (39–43)	87.2	SMD = -0.79 (-1.41, -0.17)	0.01

Subgroup analyses (**Table 3**) revealed significant effects on depression for exercise duration ≤1 h per session (SMD = -0.86, 95% CI: -1.57 to -0.16, P< 0.05, I² = 88.4%), frequency >5 sessions per week (SMD = -0.76, 95% CI: -1.12 to -0.40, P< 0.01, I² = 0%), and intervention period ≤12 weeks (SMD = -0.79, 95% CI: -1.41 to -0.17, P< 0.05, I² = 87.2%). Other parameters showed no significant differences.

#### 3.4.3 Sensitivity analysis

Sensitivity analyses evaluated the robustness of meta-analysis results. Inclusion of Wang et al. (2017) (42) significantly increased heterogeneity. Excluding this study reduced I² from 85% to 33.6%, identifying it as a primary heterogeneity source (**Table 4**).

**Table 4:** Pooled effects on depression and anxiety after excluding indivi]dual studies.

Excluded Study	SMD(95% CI)	P-value	I² (%)
Kilbourne 2012 (38)	-0.70 (-1.25, -0.15)	0.01	85.75
Zhao 2023 (39)	-0.57 (-1.15, 0.01)	0.05	86.24
Park 2021 (40)	-0.64 (-1.20, -0.09)	0.02	88.06
Sylvia 2019 (21)	-0.69 (-1.24, -0.14)	0.01	86.75
Weinstock 2016 (41)	-0.72 (-1.22, -0.21)	0.01	86.05
Wang 2017 (42)	-0.46 (-0.74, -0.18)	<0.01	33.58
Zhang 2024 (43)	-0.61 (-1.20, -0.03)	0.04	86.64

Given the small number of included studies, an assessment of publication bias was not performed (44).

### 3.5 Evidence quality assessment

GRADEPro analysis rated evidence quality as low for anxiety and very low for depression and mania (**Table 5**).

**Table 5:** GRADE evidence quality assessment.

Outcome	Included studies	Risk of bias	Inconsistency	Indirectness	Imprecision	Publication bias	Overall quality
Depression	7 (21, 38–43)	Not serious	Very Serious ^1*^	Not serious	Not serious	Undetected	**Low**
Mania	5 (21, 38, 40, 41, 43)	Not serious	Serious ^2*^	Not serious	Serious^3*^	Undetected	**Low**
Anxiety	3 (39, 40, 43)	Not serious	Serious ^2*^	Not serious	Serious^4*^	Undetected	**Low**

1.I² ≥ 75%: Considered substantial heterogeneity (downgraded by 2 levels for inconsistency). 2.50%< I²< 75%: Considered moderate heterogeneity (downgraded by 1 level for inconsistency). 3.95% CI crosses the null line: Imprecise estimate (downgraded by 1 level for imprecision). 4. Total sample size< 400 for continuous outcomes: Considered underpowered (downgraded by 1 level for imprecision).

Depression evidence was downgraded two levels due to high heterogeneity (I² = 85%).

Mania evidence was downgraded one level for heterogeneity (I² = 60.6%) and one for imprecision (95% CI crossing the null effect).

Anxiety evidence was downgraded one level for heterogeneity (I² = 71.4%) and one for imprecision (sample size<400).

### 3.6 Adverse events

No adverse events from exercise interventions were reported in the 7 studies.

## 4 Discussion

This meta-analysis of 7 studies evaluated the effects of exercise on depressive, manic, and anxiety symptoms in BD. Exercise selectively ameliorates emotional symptoms in BD, significantly reducing depressive and anxiety symptoms but not affecting manic symptoms. This aligns with meta-analyses by Schuch (11) and Heissel (45), confirming significant reductions in depressive symptoms with exercise in BD. This effect may be driven by enhanced neuroplasticity (e.g., increased BDNF release) (15). Exercise reduces anxiety symptoms by regulating HPA axis function (e.g., lowering cortisol levels) (46), consistent with studies on its anxiolytic effects. With only three studies and a small sample size for anxiety outcomes, results require cautious interpretation.

Exercise does not ameliorate manic symptoms. This contrasts with some prior studies. Wright et al. (24) reported mania-like symptoms (e.g., elevated mood, reduced sleep need) in BD patients undergoing marathon training or HIIT. In contrast, high-intensity exercise may trigger manic or hypomanic episodes by activating the sympathetic nervous system (e.g., increasing norepinephrine and dopamine) (25) and disrupting sleep rhythms (47). Low-intensity exercise regimens (e.g., yoga, qigong, walking) in this study did not exacerbate manic symptoms. Evidence on exercise effects on manic symptoms in BD remains limited, requiring further research. This may be explained by the fact that most included studies implemented interventions during the depressive phase of BD. In contrast, exercise during manic episodes presents substantial challenges due to patients’ heightened arousal, impaired judgment, and reduced adherence to structured interventions. Designing safe and feasible exercise interventions for individuals in manic states requires cautious consideration, clinical supervision, and tailored modalities such as stretching or low-intensity activities. Thus, the emotional phase of BD significantly influences the feasibility and safety of exercise interventions, and future studies should stratify participants by symptom phase when designing and evaluating exercise-based treatments.

Subgroup analyses examined optimal aerobic exercise dosage for depressive symptoms in BD across session duration, frequency, and intervention period. Regimens with session durations ≤60 min per session, frequencies >5 sessions per week, and intervention periods ≤12 weeks significantly reduced depressive symptoms. Multiple randomized controlled trials confirm significant reductions in depressive symptoms with these regimens. Firth et al. (48) reported a 32% increase in depressive symptom remission with high-frequency exercise. Schuch et al. (11) found greater depressive symptom reduction in the long-duration exercise group compared with the short-duration group, likely due to sustained cortisol regulation (49). Melo et al. (50) reported higher treatment completion rates in the short-term intervention group, potentially reflecting greater motivation for short-term goals in BD, consistent with self-efficacy theory (48). Short-term, measurable goals enhance self-efficacy in mood disorder patients (51). These findings indicate that such regimens enhance depressive symptom remission and adherence, yielding multifaceted benefits.

Limitations include: (1) Restriction to publicly available Chinese and English literature, potentially omitting relevant studies. (2) High heterogeneity from variable intervention methods, assessment tools, and patient characteristics. Inclusion of both Type I and Type II BD patients across symptomatic phases without stratified analysis may introduce bias, requiring cautious interpretation. (3) Limited study numbers, with few reporting anxiety or manic outcomes, constrain conclusion reliability due to small sample sizes and methodological variability. (4) The dose-response relationship of exercise remains unclear. Although short-duration (≤1 h per session), high-frequency (>5 sessions per week), and short-term (≤12 weeks) regimens significantly reduce depressive symptoms, other dose combinations and optimal prescription parameters require further validation. (5) The effectiveness and safety of exercise during the manic phase remains unclear. The absence of rigorous trials focusing on manic-state interventions limits the generalizability of the conclusions to all phases of BD.

Future studies should address key areas to rigorously evaluate the efficacy of exercise in BD. First, expanding literature searches, applying stricter inclusion criteria, and conducting multicenter, large-scale randomized controlled trials will enhance evidence reliability. Second, stratified analyses of Type I and Type II BD by disease phase (depressive, manic, remission) with standardized exercise parameters and assessment tools are needed. Third, researchers should explore how different emotional states affect the implementation and outcomes of exercise interventions. In particular, future studies should develop safe and effective exercise strategies specifically for the manic phase, with professional supervision and close clinical monitoring. Systematic exploration of exercise parameter combinations is required to establish dose-response models and optimize prescriptions. Long-term follow-up studies are needed to evaluate the sustained effects of exercise and its impact on BD recurrence rates. Integrating objective measures, such as imaging and biomarkers, will elucidate the neurobiological mechanisms of exercise, supporting personalized prescriptions. These advancements will provide robust evidence for standardizing exercise therapy in BD treatment.

## 5 Conclusion

This systematic review and meta-analysis suggest that exercise, an accessible and low-cost intervention, may selectively alleviate emotional symptoms in individuals with BD. Specifically, exercise appears to significantly reduce depressive and anxiety symptoms, while current evidence does not support its efficacy in reducing manic symptoms. Preliminary evidence indicates that aerobic and mind–body exercises (e.g., brisk walking, yoga), delivered in sessions of ≤1 hour, >5 times per week, and lasting no more than 12 weeks, may offer the greatest clinical benefits. However, these conclusions are drawn from studies of generally low to moderate methodological quality and should therefore be interpreted with caution. Future high-quality randomized controlled trials are needed to determine optimal exercise parameters, evaluate differential effects across BD subtypes, and establish standardized exercise protocols. These findings provide initial guidance for clinicians aiming to incorporate personalized exercise regimens as part of comprehensive BD management, while also identifying key directions for future research.

## Data Availability

The original contributions presented in the study are included in the article/**Supplementary Material**. Further inquiries can be directed to the corresponding author.
