# Supplementary material for: Exercise interventions for depressive, manic, and anxiety symptoms in bipolar disorder: a systematic review and meta-analysis
Source: Front Psychiatry. 2025 Sep 22;16:1648008. doi: 10.3389/fpsyt.2025.1648008 (PMC12497790; doi:10.3389/fpsyt.2025.1648008)
Supplement: Supplementary file 1 [file DataSheet1.pdf]

Appendix

Table 1. Systematic Literature Search Strategy

Database	Search Steps
PubMed and The Cochrane Library	#1 'Bipolar Disorder' OR 'Manic-Depressive Illness' [Title/Abstract] OR 'Bipolar Affective Disorder' [Title/Abstract] OR 'Bipolar Depression' [Title/Abstract] OR 'Manic Episode' [Title/Abstract] OR 'manic depression' [Title/Abstract]
	#2 'Exercise Therapy' OR 'Rehabilitation Exercise' [Title/Abstract] OR 'Remedial Exercise' [Title/Abstract] OR 'Exercise Intervention' [Title/Abstract] OR 'Aerobic Exercise' [Title/Abstract] OR 'Resistance Training' [Title/Abstract] OR 'Mind-Body Therapy' [Title/Abstract] OR 'Physical Activity' [Title/Abstract]OR 'Exercise' [Title/Abstract]
	#3 Randomized controlled trial [Publication Type] OR 'Randomized' [Title/Abstract] OR 'controlled' [Title/Abstract] OR 'Trial' [Title/Abstract]
	#4 #1 AND #2 AND #3
Web of Science	#1 TS= ('Bipolar Disorder' OR 'Manic-Depressive Illness' OR 'Bipolar Affective Disorder' OR 'Bipolar Depression' OR 'Manic Episode' OR 'manic depression')
	#2 TS= ('Exercise Therapy' OR 'Exercise Intervention' OR 'Aerobic Exercise' OR 'Resistance Training' OR 'Mind-Body Therapy' OR 'Physical Activity' OR Exercise OR 'Physical Activity')
	#3 TS= ('Randomized controlled trial' OR ' Randomized ' OR ' Controlled' OR 'Trial')
	#4 #1 AND #2 AND #3
Embase	#1 'bipolar disorder' /exp OR 'bipolar disorder' OR 'manic-depressive illness' :ab, ti OR 'bipolar' OR 'bipolar depression :ab, ti OR 'manic episode' :ab, ti OR 'manic depression' :ab, ti

	#2 ‘exercise’/exp OR ‘exercise’ OR ‘functional training’:ab, ti OR ‘kinesiotherapy’/exp OR ‘kinesiotherapy’ OR ‘exercise therapy’:ab, ti OR ‘physical activity’exp OR ‘physical activity’:ab, ti OR ‘rehabilitation exercise’:ab, ti OR ‘remedial exercise’:ab, ti OR ‘exercise intervention’/exp OR ‘exercise intervention’:ab, ti OR ‘aerobic exercise’/exp OR ‘aerobic exercise’:ab, ti OR ‘resistance training’/exp OR ‘resistance training’:ab, ti OR ‘mind-body therapy’:ab, ti<br>#3 ‘Randomized controlled trial’[exp] OR ‘Randomized’[ab, ti] OR ‘Controlled’[ab, ti] OR ‘Trial’[ab, ti]<br>#4 #1 AND #2 AND #3<br>#5 #4 AND ‘bipolar disorder’/dm AND (‘human’/de OR ‘randomized controlled trial’/de) |
CNKI	主题=（双相情感障碍 + 双相情感障碍患者+ 情绪障碍 + 躁郁症 + 双相障碍 + 双相抑郁 + 躁狂发作 +轻躁狂） AND 主题=（运动疗法 + 运动疗法干预 + ‘运动疗法/方法’ + 运动 + 运动干预 + 体育锻炼 + 有氧运动 + 抗阻训练 + 身心疗法 + 体力活动
Wan fang and VIP	主题=（双相情感障碍 OR 双相情感障碍患者 OR 情绪障碍 OR 躁郁症 OR 双相障碍 OR 双相抑郁 OR 躁狂发作 OR 轻躁狂） AND 主题=（运动疗法 OR 运动疗法干预 OR 运动干预 OR 运动 OR 体育锻炼 OR 有氧运动 OR 抗阻训练 OR 身心疗法 OR 体力活动）
